# Supplementary figures and images for: Mimulus sRNAs Are Wound Responsive and Associated with Transgenerationally Plastic Genes but Rarely Both
Source: Int J Mol Sci. 2020 Oct 13;21(20):7552. doi: 10.3390/ijms21207552 (PMC7589798; doi:10.3390/ijms21207552)

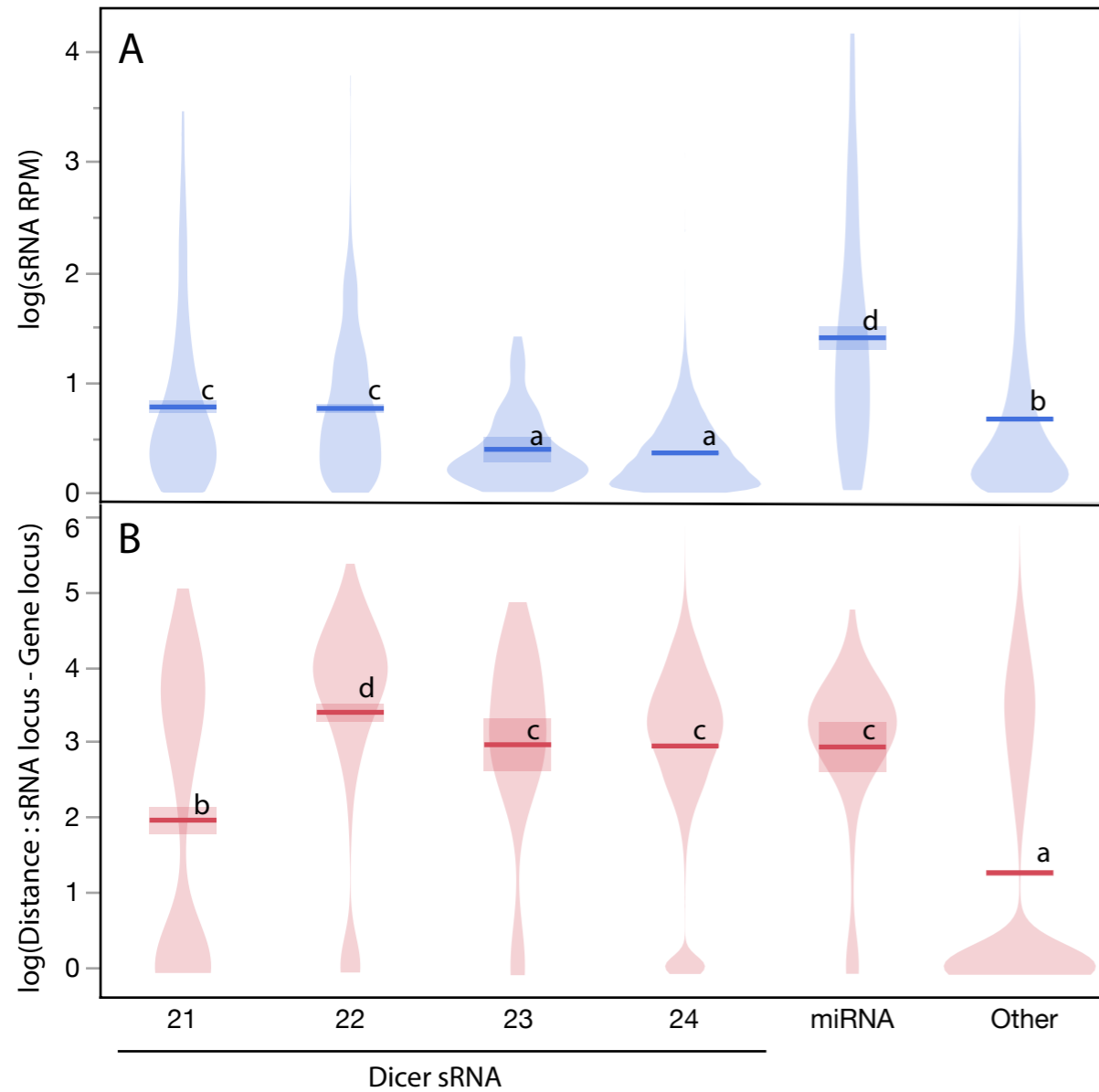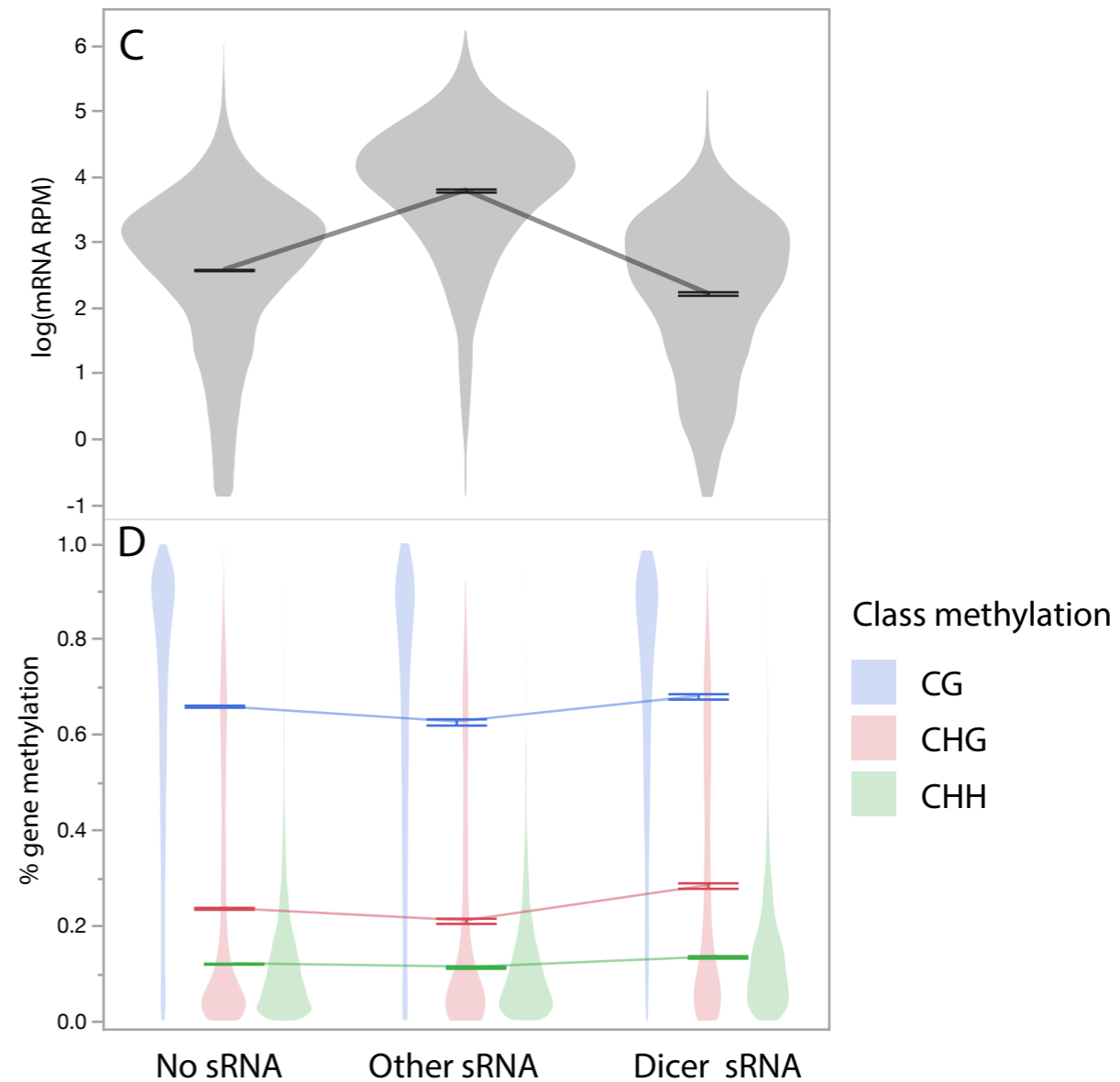

Supplement: Supplementary file 1 [file ijms-21-07552-s001.zip › Figure_S1F.pdf]
